# Predominant but silent C1q deposits in mesangium on transplanted kidneys - long-term observational study

**DOI:** 10.1186/s12882-018-0874-9

**Published:** 2018-04-06

**Authors:** Takahiro Kanai, Yuko Akioka, Kenichiro Miura, Masataka Hisano, Junki Koike, Yutaka Yamaguchi, Motoshi Hattori

**Affiliations:** 1 Department of Pediatric Nephrology, Kidney Center, Tokyo Women’s Medical University, Tokyo, Japan; 2 Department of Pediatrics, Jichi Medical University, 3311-1 Yakushiji, Shimotsuke, Tochigi 329-0498 Japan; 3 Department of Pediatrics, Saitama Medical University, Moroyama, Saitama Japan; 4 Department of Pathology, Kawasaki City Tama Hospital, Kawasaki, Kanagawa Japan; 5 Yamaguchi’s Pathology Laboratory, Chiba, Japan

**Keywords:** C1q nephropathy, C1q deposits, Clinicopathologic study, Protocol renal graft biopsy, Renal transplantation

## Abstract

**Background:**

C1q nephropathy (C1qN) was first described as glomerular disease characterized by predominant meangial C1q deposits in patients with proteinuria and no evidence of systemic lupus erythematosus. Several studies, however, revealed the clinical heterogeneity of C1qN, showing some cases with normal urinalysis. To confirm the existence of cases with predominant mesangial C1q deposits and negative or mild proteinuria and/or hematuria, we investigated renal graft biopsy specimens showing negative to mild proteinuria (less than or equal to 1+ by dip stick test) and/or hematuria.

**Methods:**

Eligible participants were kidney transplant cases who corresponded to the criteria for C1qN and were followed more than 10 years. Their medical records were reviewed to determine the age at detection of predominant mesangial C1q deposits, gender, original renal disease and reason for renal graft biopsy, blood pressure, degree of proteinuria and hematuria, and serum creatinine levels.

**Results:**

From 414 cases in adults and children, five pediatric patients (the male to female ratio, 1:1.5) were eligible. At the time when predominant mesangial C1q deposits were detected, 2 cases presented with mild proteinuria without hematuria, but the other 3 cases showed normal urinalysis. Light microscopy revealed minor glomerular abnormality in all the cases. Immunofluorescent study showed predominant mesangial C1q deposits with IgG, IgM and C3 in all cases. All selected specimens presented electron dense-depos in the mesangium. Ten years later from the detection, 2 cases continued to be normal urinalysis and 3 cases had mild proteinuria without hematuria. During this follow-up period, no cases presented with persistent proteinuria and/or hematuria greater than or equal to 2+ by dip stick test. And no cases developed systemic lupus erythematosus. Follow-up renal graft biopsies were performed once in 2 cases 8 years later from the detection. They showed minor glomerular abnormalities. C1q deposit disappeared in one case. In another case, immunofluorescent study was not examined.

**Conclusions:**

This long-term observational study on transplanted kidneys confirms the existence of cases with predominant but silent C1q deposits in the mesangium who have negative or mild proteinuria.

**Electronic supplementary material:**

The online version of this article (10.1186/s12882-018-0874-9) contains supplementary material, which is available to authorized users.

## Background

C1q nephropathy (C1qN) was described by Jennette and Hipp in 1985 [1, 2]. It is characterized by the following criteria: (i) the presence of predominant or co-dominant mesangial C1q deposits detected by immunofluorescence microscopy (IF), (ii) corresponding mesangial or para-mesangial electron-dense deposit (EDD), and (iii) absence of clinical and serological evidence of systemic lupus erythematosus (SLE). Clinically, patients have proteinuria in the nephrotic range and it is resistant to steroid treatment.

Clinicopathologic findings of C1qN have accumulated from various reports [3–5]. One study identified some cases with normal urinalysis and predominant mesangial C1q deposits [6].

To confirm the existence of cases with predominant mesangial C1q deposits and negative to mild proteinuria and/or hematuria, we investigated cases with renal graft biopsied specimens who had negative to mild proteinuria and/or hematuria.

## Methods

### Selection criteria

We investigated 414 serial cases with negative to mild proteinuria (less than or equal to 1+ by dip stick test) and/or hematuria (less than or equal to 1+ by dip stick test) who received renal transplantations. Renal graft biopsy specimens from cases were obtained between 2002 and 2005 at the Kidney Center in Tokyo Women’s Medical University. The cases consisted of 334 adults aged 19 years old or older, and 80 children aged 2 to less than or equal to 18 years. There were 242 males and 172 females.

Cases were selected if they had predominant mesangial C1q deposits (2+ or greater on a ± 4 scale) by the definition of C1qN [1, 2, 6], and if the follow-up period was more than 10 years since the predominant C1q deposits in the mesangium were detected.

We excluded cases with morphologic features of membranoproliferative glomerulonephritis type I [1, 6] or that fulfilled the diagnostic criteria for SLE [7] because they show mesangial C1q deposits.

### Clinical data and laboratory results

The patients’ medical records were reviewed, noting the age at detection of predominant mesangial C1q deposits, gender, original renal disease, reason for renal graft biopsy, and blood pressure levels. The laboratory results for the following tests were also reviewed: degree of proteinuria and hematuria, anti-C1q antibody titers, and serum creatinine levels. Additionally, data on medications was collected including use of steroids, immunosuppressants, angiotensin receptor blockers (ARBs), and angiotensin converting enzyme inhibitors.

### Pathologic findings

All specimens were examined by light microscopy (LM; hematoxylin-eosin, periodic acid-Schiff, and periodic-methenamine silver), IF (polyclonal rabbit anti-human IgG, IgM, IgA, C1q, and C3 antibody, Dako, Denmark), and electron microscopy (EM) according to standard techniques. Each glomerular tissue specimen was available for all three methods of evaluation for the detection of predominant mesangial C1q deposits. The degree of IF staining was graded as − (negative), ± (trace), 1+ (mild), 2+ (moderate), 3+ (marked), and 4+ (heavy) according to Jennette’s criteria [1, 2, 6].

## Results

### Eligible cases, clinical data, and laboratory results

Five pediatric patients (the male to female ratio, 1:1.5) at selection met the criteria for C1qN [1, 2, 6]. Samples from these 5 patients constituted 0.01% of all samples investigated.

The original renal diseases consisted of renal hypoplasia, focal segmental glomerulosclerosis (FSGS), autosomal recessive polycystic kidney disease, Alport syndrome, and chronic tubulointerstitial nephritis. All 5 patients received kidney transplantation (kidneys donated by their living parents) at a mean age of 8.8 years (SD 2.2). Reasons for renal graft biopsies at the time when predominant C1q deposits were detected were due to protocol in 4 cases, and mild proteinuria in 1 case (Table 1). Protocol renal graft biopsies were carried out an average of 2.2 times (range, 1 to 3 times) before predominant mesangial C1q deposits were detected. No case presented with hypertension [8] or signs or symptoms of infection when predominant mesangial C1q deposits were detected. The follow-up period after the detection of predominant C1q deposits ranged from 10 to 11 years.

**Table 1:** Clinical and pathologic findings at the time when predominant mesangial C1q deposits were detected

Case No.	Reason for RBx	Laboratory results			LM findings	IF findings					EM findings			
		Up	OB	sCr [mg/dL]		IgG	IgM	IgA	C1q	C3	Mes	Subend	Subep	ARB
1	Protocol	(−)	(−)	0.7	MGA	2+	1+	1+	2+	1+	(+)	(−)	(+)	–
2	Protocol	(+)	(−)	0.6	MGA	2+	1+	–	2+	1+	(+)	(−)	(−)	losartan potassium 100 mg/day
3	Protocol	(−)	(−)	1.2	MGA	2+	1+	–	2+	1+	(+)	(+)	(−)	–
4	Mild proteinuria	(+)	(−)	0.7	MGA	2+	2+	–	2+	1+	(+)	(−)	(−)	–
5	Protocol	(−)	(−)	0.6	MGA	1+	1+	–	2+	1+	(+)	(−)	(+)	losartan potassium 25 mg/day

*Abbreviation: No* number, *RBx* renal biopsy, *LM* light microscopy, *IF* immunofluorescent, *EM* electron microscopy, *Up* urinary protein, *OB* occult blood, *sCr* serum creatinine, *MGA* minor glomerular abnormality, *Mes* mesangial deposits, *subend* subendothelial deposits, *Subep* subepithelial deposits

When predominant mesangial C1q deposits were detected, 2 cases presented with mild proteinuria (1+ by dipsticks) without hematuria, but the other 3 cases showed normal urinalysis results (Table 1).

All eligible cases had anti-C1q antibody titers in the normal range (reference anti-C1q antibody titer, less than 2.2 μg/mL) [9]. 4 cases had normal serum creatinine levels and stable renal function. One case had a moderate increase in serum creatinine level (from 0.5 to 1.2 mg/dL) due to acute rejection 11 months after renal transplantation. After that, the patient maintained stable renal function.

During the follow-up period, no cases presented with persistent proteinuria and/or hematuria greater than or equal to 2+ by dip stick test. Additionally, no cases presented with hypertension or developed SLE.

These 5 patients were given immunosuppressive agents against rejection as follows: tacrolimus hydrate (adjusted to target level of 3–5 ng/mL), mycophenolate mofetil (700 mg/m^2^/day), and methylprednisolone (1 to 4 mg/day).

At the time of diagnosis, 2 cases had received an ARB, losartan potassium, at 100 mg/day or 25 mg/day, respectively. All cases had received ARBs 10 years after the predominant C1q deposits were detected (Table 2). No cases had received angiotensin converting enzyme inhibitors at the time of detection of predominant mesangial C1q deposits or 10 years later.

**Table 2:** Clinical and pathologic findings 10 years later

Case No.	Laboratory results			LM findings	IF findings	ARB
	Up	OB	sCr [mg/dL]			
1	1+	–	1.27	MGA(8 y later)	NA	candesartan cilexetil 25 mg/day
2	1+	–	1.39	NA	NA	losartan potassium 100 mg/day
3	–	–	1.78	MGA (8 y later)	Negative for C1q, IgG, IgM, IgA and C3	losartan potassium 25 mg/day
4	±	–	0.83	NA	NA	valsartan 60 mg/day
5	–	–	0.80	NA	NA	losartan potassium 37.5 mg/day

*Abbreviation: No* number, *LM* light microscopy, *IF* immunofluorescent, *Up* urinary protein, *OB* occult blood, *sCr* serum creatinine, *MGA* minor glomerular ab normality, *NA* not available, *ARB* angiotensin receptor blocker

### Pathologic findings

Details of LM, IF EM findings are presented in Table 1.

**Fig. 1:**
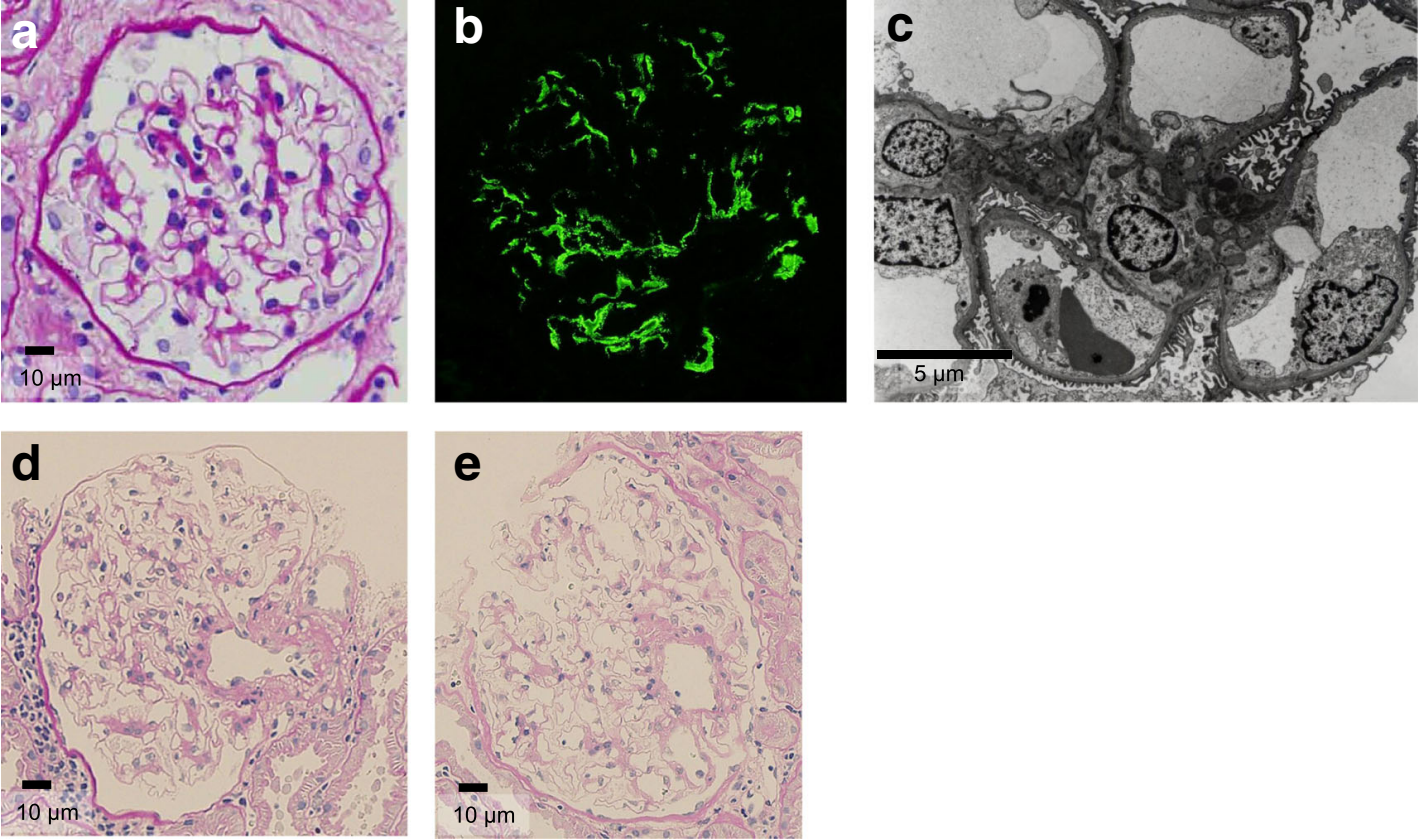
Pathologic findings. **a** Minor glomerular abnormality. Periodic acid-Schiff stain. Original magnification × 600. **b** Immunofluorescence detection of C1q deposits in mesangial areas (2+ intensity). **c** Electron-dense deposits on mesangial and para-mesangial areas. **d** and **e** Minor glomerular abnormality. Periodic acid-Schiff stain. Original magnification × 200

Averages of 28 glomeruli (10 to 63 glomeruli) were observed with LM; all these glomeruli showed minor glomerular abnormalities (MGA) (Table 1, Fig. 1a).

IF revealed predominant mesangial C1q deposits and co-deposits (Table 1, Fig. 1b). C1q deposits were limited to the mesangium or para-mesangium areas, except for 1 case whose specimen also exhibited staining in some capillary loops. Equal or less intense mesangial IgG, IgM, and C3 deposits were detected in all selected specimens (Table 1). IgA deposits were observed only in 1 case.

All selected specimens showed EDDs in the mesangium on EM (Table 1, Fig. 1c). Subendothelial deposits were observed in 1 case and subepithelial deposits were observed in 2 cases. No specimens showed tubuloreticular inclusions or foot process effacements.

Two cases had biopsies of their native kidneys before renal transplantation, but did not present with mesangial C1q deposits and EDDs. Renal graft biopsy specimens from transplanted kidneys at renal transplantation in these 5 cases were absent of C1q deposits and EDDs.

In 2 cases, follow-up renal graft biopsies after C1q detection on mesangium were performed once 8 years later in case (Table 2). Their glomeruli presented with MGA in the LM studies. The IF study in 1 case was negative for C1q, IgG, IgM, IgA, and C3 (Table 2, Fig. 1d and e). In another case, the IF study was not examined.

## Discussion

This long-term observational study on transplanted kidneys confirms the existence of cases with predominant but silent C1q deposits in the mesangium who have negative or mild proteinuria with MGA.

Said et al. reported that patients who had predominant mesangial C1q deposits with MGA on their transplanted kidneys, had maintained no proteinuria for a mean follow-up of 1 year [10]. Our study, moreover, demonstrated that patients who had predominant mesangial C1q deposits with MGA on their transplanted kidneys, had maintained negative to mild proteinuria without hematuria even for 10 years follow-up.

### Differences between our eligible cases and previous reports of C1qN

The age at diagnosis and gender ratio were similar to those of previous reports of C1qN [1, 2, 4]. Pathologically, the LM, IF, and EM findings also corresponded to those in Jennette’s reports (Table 1) [1, 2]. EM showed a similar rate of occurrence of subendothelial and subepithelial EDDs in our eligible cases as in those of Jennette’s reports [1, 2, 4].

The prevalence of C1qN differs in various reports, ranging from 0.2 to 1.9% in biopsies from children and adults [2, 6]. The 0.01% incidence in our sample population was less than the incidence of C1qN in previous reports. The reason might be that our study was done in cases with transplanted kidneys. Renal graft biopsy is performed repeatedly by protocol, even in patients with normal urinalysis.

No cases showed FSGS or mesangial proliferative glomerulonephritis (MesPGN) in our study. As for IgA deposits, only 1 out of the 5 cases had positive staining, although 9 cases out of 15 exhibited positive staining in Jennette’s previously published case series [1, 2]. In this regard, our eligible cases differ from those in previous reports of C1qN. These differences might be related to the negative or mild proteinuria or hematuria observed in our cases.

### Why was normal urinalysis observed in our eligible cases?

Immune complex deposits are thought to cause nephritis by attracting and activating intrinsic glomerular cells to release local mediators of inflammation [11]. On the other hand, one report suggested that overt C1qN would require additional factors in addition to C1q deposits. Issac et al. suggested that BK polyoma viral infection could trigger C1qN [12], although our eligible cases were not positive for BK polyoma viral infection as assessed by SV40 T antibody staining in any renal graft specimens.

C1q deposits might be only innocent bystanders. Waldherr et al. reported that patients with IgAN are only the tip of the iceberg among patients with IgA deposits in their kidneys because mesangial IgA deposits are found in approximately 4.8% of the general population, whereas IgAN was found in 0.02% of the general population [13]. Thus, the prevalence of predominant but silent C1q deposits would be higher than that of C1qN.

Although C1qN was previously described as MGA, FSGS, and MesPGN in LM [1–5], no eligible cases in the current study presented with FSGS or MesPGN. This might mean that our eligible cases did not have C1qN, but only silent C1q deposits, similar to silent predominant mesangial IgA deposits that present in MGA [13]. This would also explain the reason for the negative to mild proteinuria findings in contrast to previous reports of C1qN [1–5].

Another possibility for why our eligible cases did not experience abnormal urinalysis is that immunosuppressive therapy against rejection might suppress the inflammation, although Kersnik Levart et al. reported that cyclosporine A and/or mycophenorate mofetil were not effective for some cases of C1qN [14].

ARBs could prevent proteinuria; however, 2 cases did not experience proteinuria before ARBs were administrated. Moreover, in one of the 2 cases, not only C1q deposits but all other deposits were not detected 8 years later. This suggests the existence of cases with predominant but silent C1q deposits in the mesangium.

## Conclusion

This long-term observational study on transplanted kidneys confirms that there are cases with predominant but silent C1q deposits in the mesangium who have negative or only mild proteinuria with MGA. This provides a first step towards a better understanding of the role of predominant mesangial C1q deposits.

## Additional file

Additional file 1: Pathologic findings. Minor glomerular abnormalities stained with Periodic acid-Schiff stain. Original magnification × 600. Immunofluorescence detection of C1q deposits in mesangial areas (2+ intensity). Electron-dense deposits on mesangial and para-mesangial areas. Minor glomerular abnormality. (PPTX 8948 kb)

## Abbreviations

ARB: Angiotensin receptor blocker
C1qN: C1q nephropathy
EDD: Electron-dense deposit
EM: Electron microscopy
FSGS: Focal segmental glomerulosclerosis
IF: Immunofluorescence microscopy
LM: Light microscopy
MesPGN: Mesangial proliferative glomerulonephritis
MGA: Minor glomerular abnormality
SLE: Systemic lupus erythematosus
