# Supplementary figures and images for: Predominant but silent C1q deposits in mesangium on transplanted kidneys - long-term observational study
Source: BMC Nephrol. 2018 Apr 6;19:82. doi: 10.1186/s12882-018-0874-9 (PMC5889604; doi:10.1186/s12882-018-0874-9)

## Slide 1
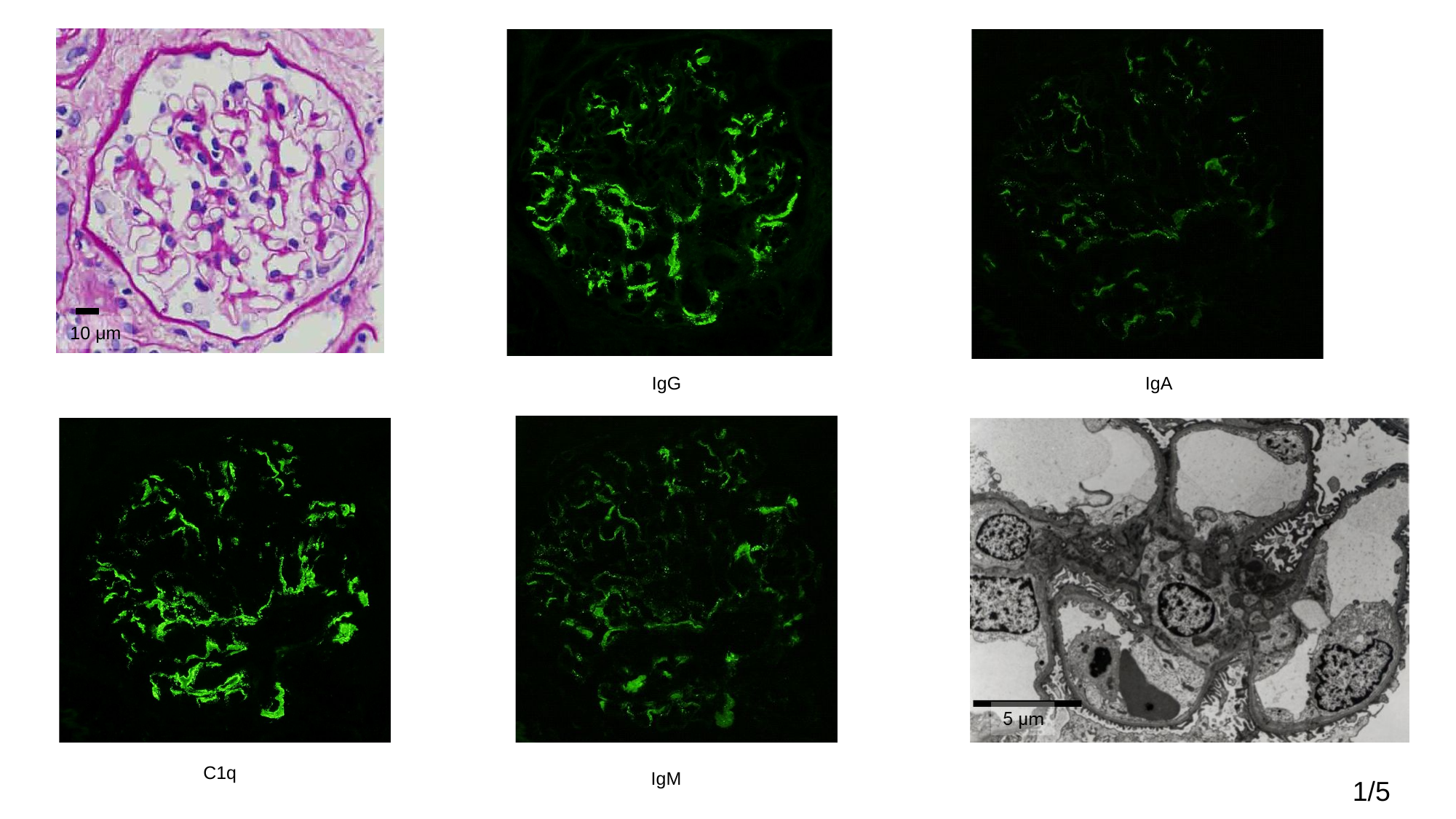

10 μm
IgG
IgA
C1q
IgM
1/5

## Slide 2
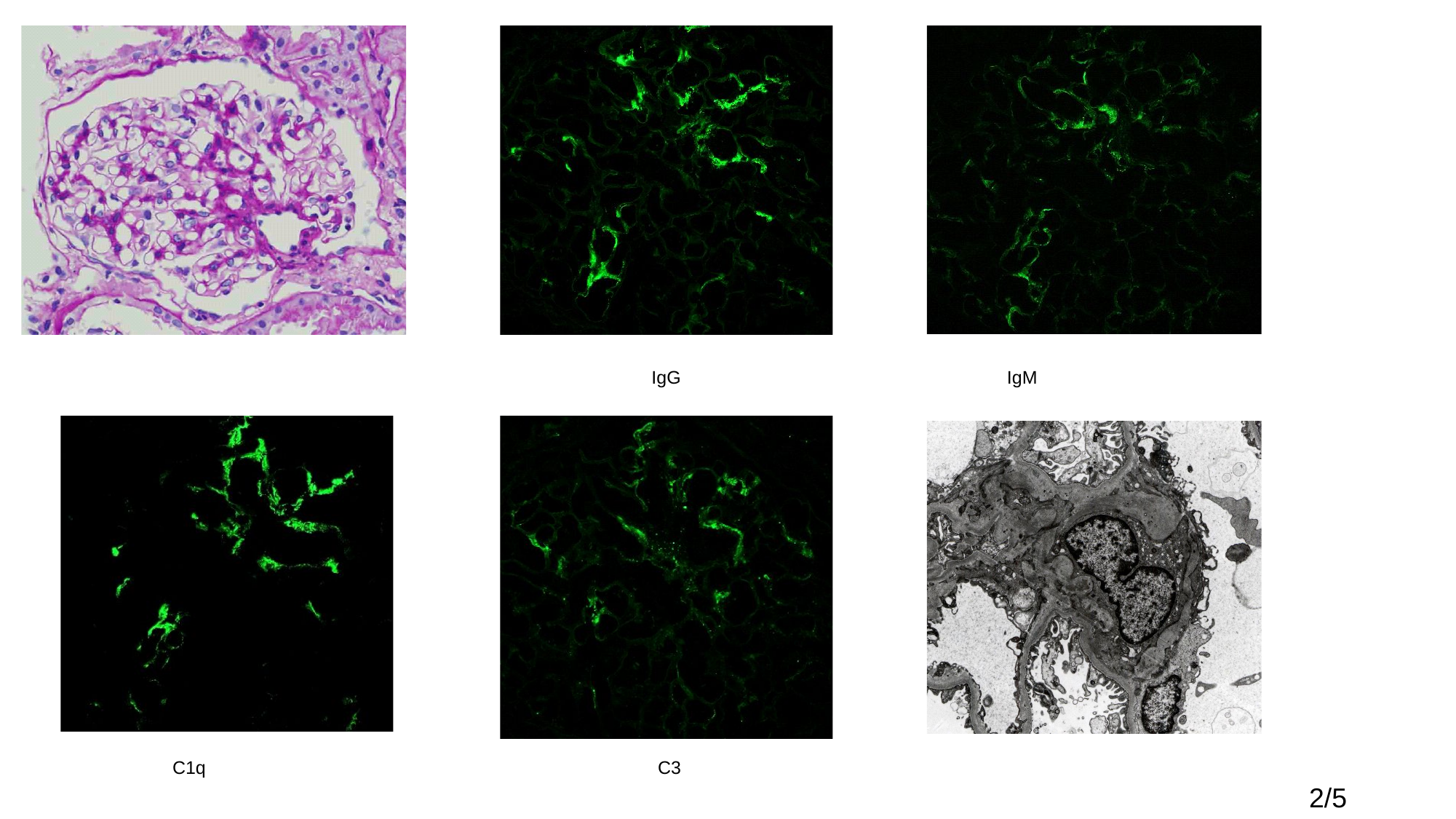

IgG
IgM
C1q
C3
2/5

## Slide 3
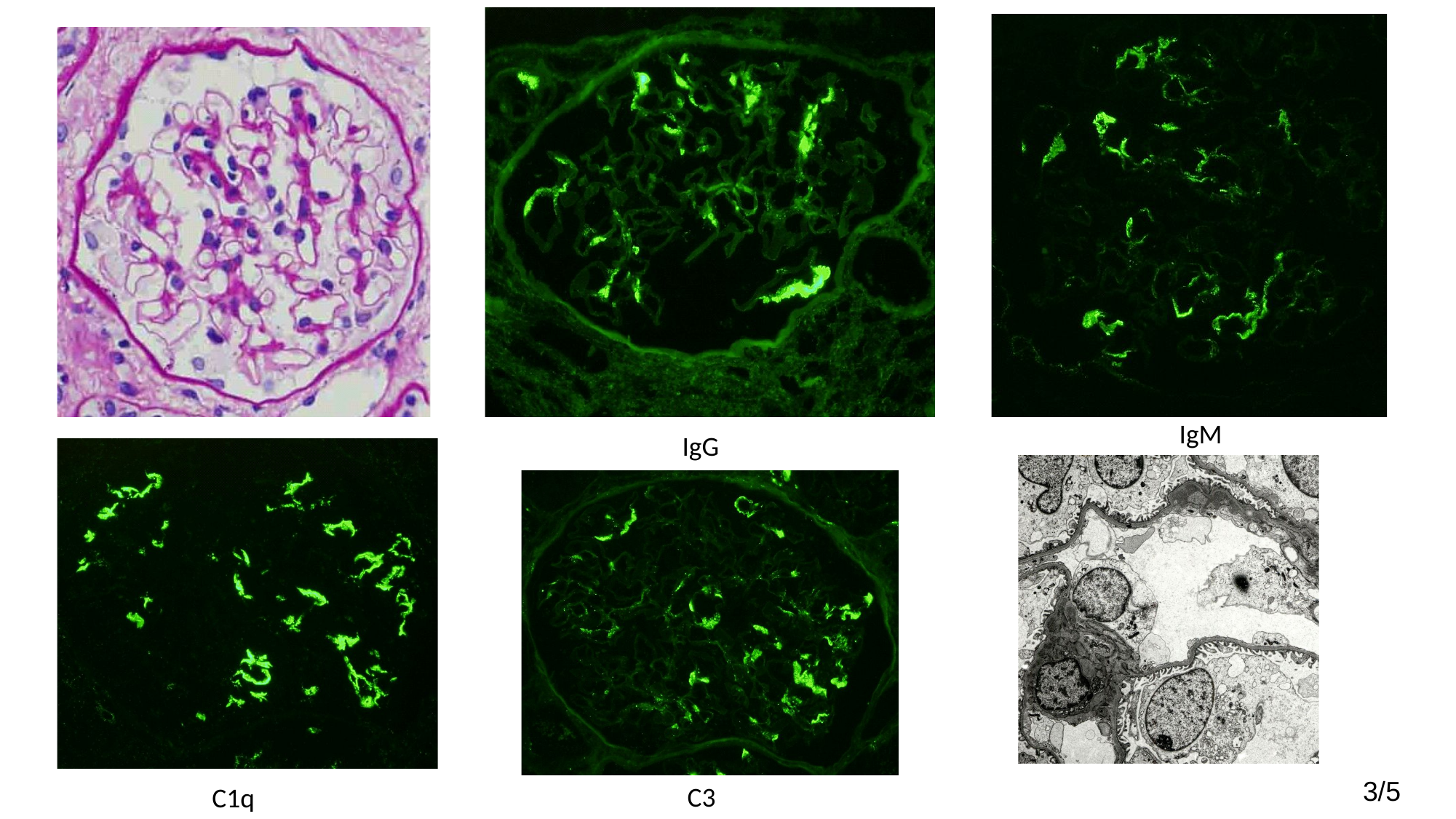

IgM
IgG
3/5
C3
C1q

## Slide 4
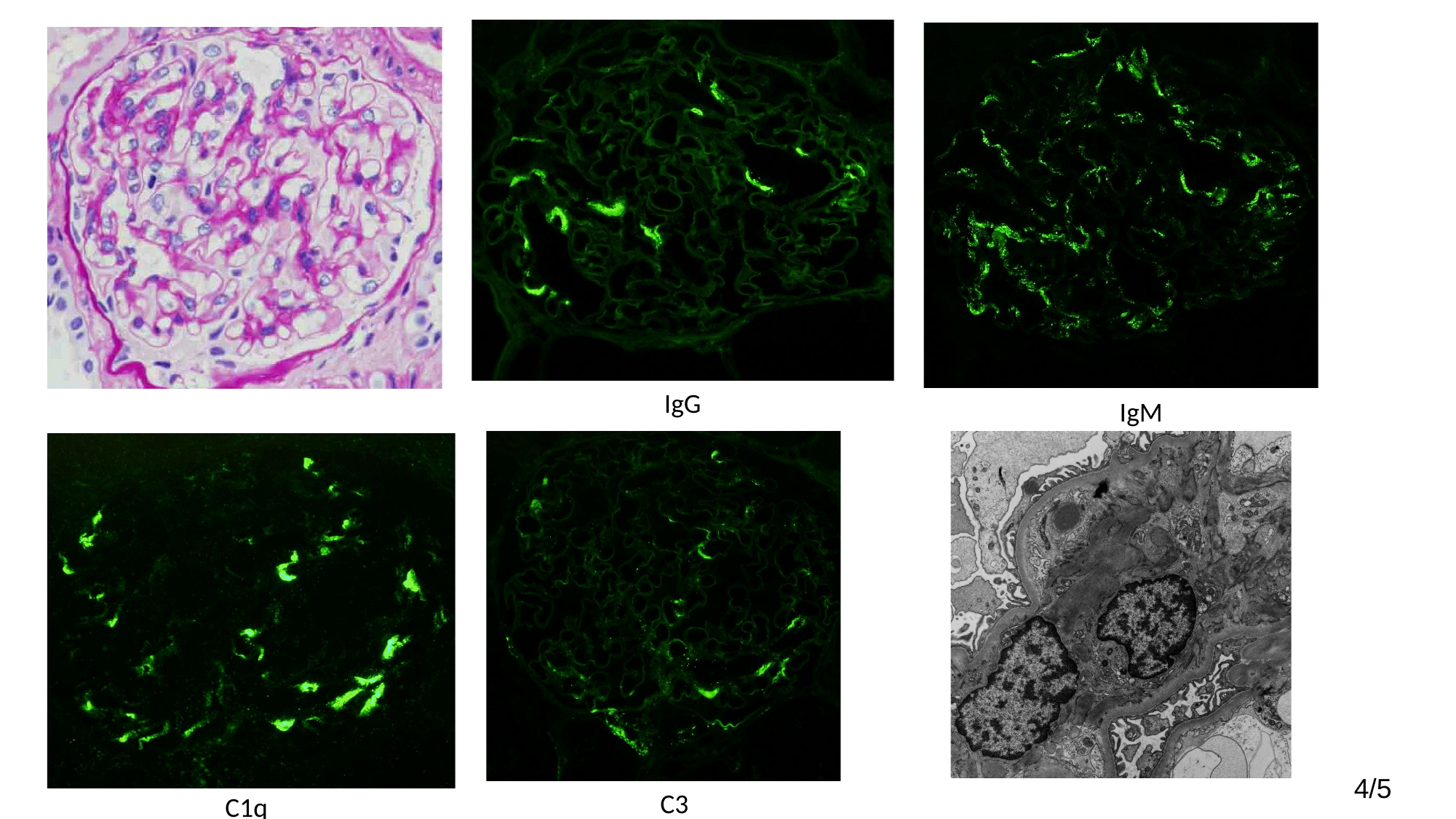

IgG
IgM
4/5
C3
C1q

## Slide 5
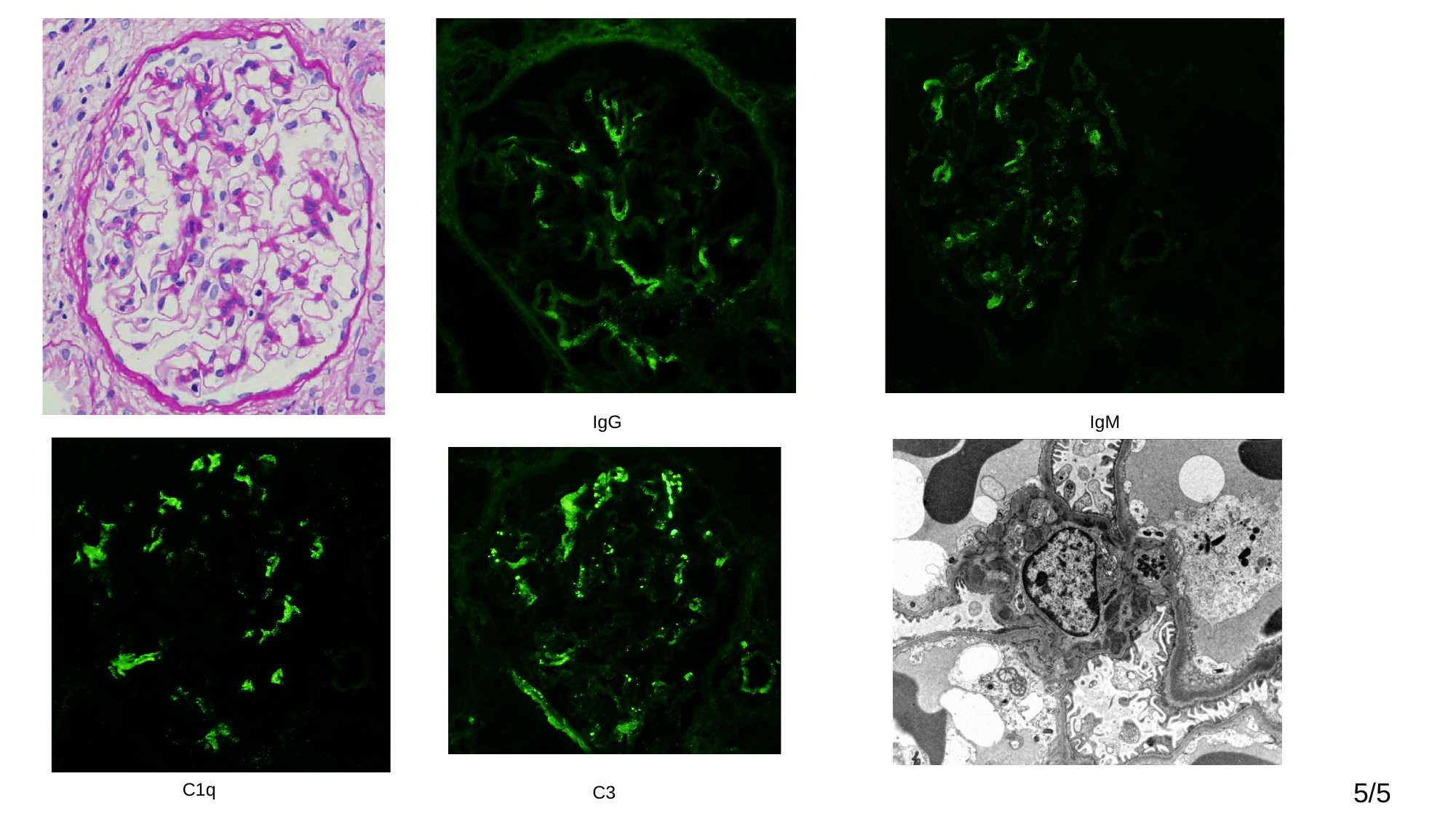

IgM
IgG
5/5
C1q
C3

Supplement: Supplementary file 1 — Pathologic findings. Minor glomerular abnormalities stained with Periodic acid-Schiff stain. Original magnification × 600. Immunofluorescence detection of C1q deposits in mesangial areas (2+ intensity). Electron-dense deposits on mesangial and para-mesangial areas. Minor glomerular abnormality. (PPTX 8948 kb) [file 12882_2018_874_MOESM1_ESM.pptx]
